# Prevalence of mental disorders and epidemiological associations in post-conflict primary care attendees: a cross-sectional study in the Northern Province of Sri Lanka

**DOI:** 10.1186/s12888-019-2064-0

**Published:** 2019-03-04

**Authors:** Shannon Doherty, E. Hulland, B. Lopes-Cardozo, S. Kirupakaran, R. Surenthirakumaran, S. Cookson, C. Siriwardhana

**Affiliations:** 1 Faculty of Health, Education, Medicine, and Social Care, Anglia Ruskin University, Bishop Hall Lane, Chelmsford, CM1 1SQ UK; 2 Centers for Disease Control and Prevention, 1600 Clifton Rd NE, Atlanta, GA 30329 USA; 3 THEME Institute, 81/7 Pagoda Rd, Nugegoda, Colombo, Sri Lanka; 4 University of Jaffna, Thirunelvely, Jaffna, 70140 Sri Lanka; 5 Centers for Disease Control and Prevention, 1600 Clifton Rd NE, Atlanta, GA 30329 USA; 6 London School of Hygiene and Tropical Medicine, Keppel St, London, WC1E 7HT UK

**Keywords:** Mental disorder, Conflict-affected population, Primary care, Sri Lanka

## Abstract

**Background:**

Experiencing conflict and displacement can have a negative impact on an individual’s mental health. Currently, prevalence of mental health disorders (MHDs) at the primary care level in post-conflict areas within the Northern Province of Sri Lanka is unknown. We aimed to explore this prevalence in conflict-affected populations attending primary care, using a structured package of validated screening tools for MHDs.

**Methods:**

This cross-sectional study aimed to determine factors related to mental health disorders at the primary care level in Northern Province, Sri Lanka. A structured interview was conducted with internally displaced adults attending 25 randomly selected primary care facilities across all districts of Northern Sri Lanka (Jaffna, Mannar, Mullaitivu, Vavuniya). Participants were screened for depression, anxiety, psychosis, PTSD, and somatoform symptoms.

**Results:**

Among 533 female and 482 male participants (mean age 53.2 years), the prevalence rate for any MHD was 58.8% (95% CI, 53.8–61.4), with 42.4% screening positive for two or more disorders (95% CI, 38.6–46.1). Anxiety prevalence was reported at 46.7% (95% CI, 41.9–51.5), depression at 41.1% (95% CI, 38.7–44.5), PTSD at 13.7% (95% CI, 10.6–16.8), somatoform symptoms at 27.6% (95% CI, 23.6–31.5), and psychosis with hypomania at 17.6% (95% CI, 13.3–21.9).

**Conclusion:**

This is the first study at the primary care level to investigate prevalence of MHDs among conflict-affected populations in the Northern Province, Sri Lanka. Results highlight unmet mental health needs in the region. Training intervention to integrate mental health services into primary care is planned.

## Background

Conflict-associated mental health disorders (MHDs) vary by prevalence across countries and cultures [1–3]. In low-resource settings, the impact of conflict on affected populations is compounded by low prioritization of mental health, and lack of access and integration of mental health into primary health care. Currently, the prevalence of MHDs in post-conflict Sri Lanka is unknown.

The Sri Lankan Civil War (1983–2009) resulted in an estimated 500,000 internally displaced persons (IDPs). Previous epidemiological studies have explored the burden of MHDs linked to the conflict, but were limited to community samples assessing depression, anxiety, psychosis, or Posttraumatic Stress Disorder (PTSD) [4–10]. Only one 2013 cross-sectional study, limited to 16 primary care facilities in four (of five) districts, reported prevalence of major (4.5%; 95% CI, 4.1–4.9%) and minor (13.3%; 95% CI, 12.7–13.9%) depression [5]. Thus, little is known about prevalence and predictors of MHDs at the primary care level or how to address unmet mental health needs of primary care attendees in the post-conflict region.

As there is a lack of specialized psychiatric services in the post-conflict region of Northern Province, Sri Lanka, it is vital to understand the prevalence of mental health disorders presenting at the primary care level and to understand the various factors that can increase the risk of developing mental health disorders. Knowledge gained on the unmet mental health needs of the post-conflict population could help reduce the mental health treatment gap in the region. In order to reduce the gap and enhance integration of mental health into primary care, a five-year program is being implemented across Sri Lanka’s Northern Province. This program is based on a 2015 pilot study there, completed by members of the current research team, which highlighted logistical and feasibility barriers to integration of mental health services into primary care in the region [6]. This current program, titled ‘Integrating mental health into primary care for conflict-affected populations in Northern Sri Lanka (COMGAP-S)’ has two phases. Phase One’s primary aim is to conduct an epidemiological survey at the primary care level to describe prevalence of MHDs, including depression, anxiety, somatoform disorders, PTSD, and psychosis among the conflict-affected adult internally displaced persons (IDPs) attending primary care facilities in the Northern Province. Informed by findings from Phase One, as reported in this manuscript, the primary aim of Phase Two is to integrate mental health services into primary care via a scaled-up training intervention based on the World Health Organization mental health gap intervention guide 2.0 (WHO mhGAP-IG 2.0). This paper presents the results of the Phase One study, which indicate high prevalence of unmet MHDs in adults presenting in primary care facilities in Northern Province, Sri Lanka.

## Methods

The aim of this cross-sectional study was to carry out an epidemiological survey to estimate prevalence of MHDs among adults attending primary care facilities located in all five districts (Jaffna, Mannar, Mullaitivu, Vavuniya, Kilinochchi) of Northern Province, Sri Lanka.

Inclusion criteria were both sexes, 18 years and older, internally displaced due to conflict, attending public primary care facilities, either for the first time or as a follow-up visit. Children younger than 18 and people with severe mental illness or hearing or speech disability whose conditions prevented administration of the study questionnaire were excluded.

Sample size was calculated using a prevalence of anxiety or depression - the most common MHDs - of 50.0%, a conservative value chosen based on doubling previous study prevalence conducted in Jaffna, Mannar, and Kilinochchi districts of Northern Province of depression at 22.2% (95% CI, 18.2–26.5%) and anxiety at 32.6% (95% CI, 28.5–36.9%) [7]. Due to the conservative choice of 50% prevalence, this sample size should be adequate to detect higher prevalence of concurrent anxiety conditions as well with high precision. To achieve ±5% precision, considering a 95% confidence interval, a total population of 1,234,932 in the Northern Province, an estimated design effect (DEFF) of 2.2 based on previous literature, and an assumed response rate of 80.0%, the total required sample size was calculated to be 1025 [11].

Primary sampling units were public primary care facilities in Northern Province, which included divisional hospitals and primary medical care units. The most recent data (from 2013/14) details Northern Province had 54 reported divisional hospital, 35 reported primary medical care units, and the provincial number of outpatients in district hospitals and primary medical care units were 1,445,675 and 325,480 respectively [12].

**Fig. 1:**
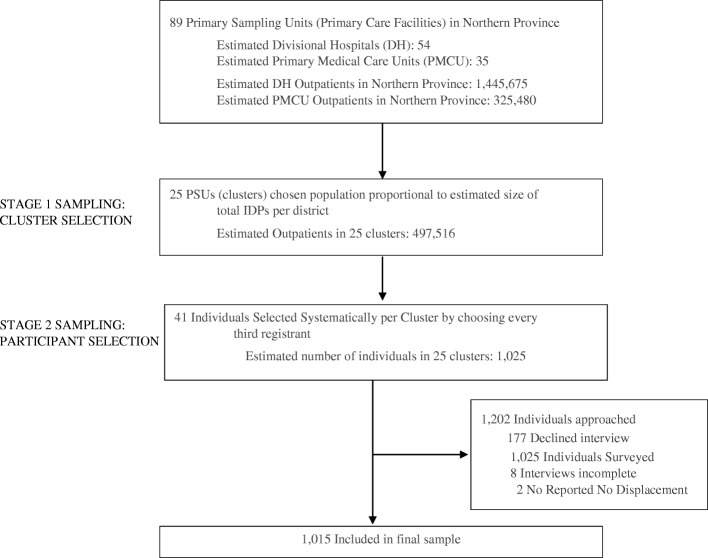
Sampling Selection

A list of public primary care facilities in all Northern Province districts was compiled by type, and 25 facilities (clusters) were randomly selected. A flowchart of the study sample is presented in Fig. 1. Distribution of clusters was allocated proportionally to total number of IDPs in each district; districts with smaller number of IDPs were assigned fewer clusters. Allocation of clusters was to account for population displacement during the last stage of the conflict in 2009, and parts of the province, which experienced less displacement, but had larger populations. This strategy ensured an adequate representation of conflict severity and displacement typology and length. From each facility (cluster), 41 individuals were recruited by systematically selecting every third attendee from the facility registration desk.

Information was gathered using a structured interview covering demographic and socio-economic background, conflict and displacement experiences, mental disorder screening, social support/networks, and health service use. MHD screening tools utilized were: Hopkins Symptom Checklist-25 (HSCL-25) for depression and anxiety, Patient Health Questionnaire (PHQ-15) for expression of somatoform symptoms, Harvard Trauma Questionnaire (HTQ) for PTSD, Psychosis Screening Questionnaire (PSQ) for psychosis. The Lubben Social Network Scale (LSN) and Multidimensional Support Scale (MDSS) were used to assess social support and social networks. The threshold for each MHD was determined by the validated scoring process for each measurement utilised. This process is detailed below.

Most instruments had been previously used in a number of epidemiological studies in Sri Lanka, especially among conflict-affected populations including IDPs [7–9, 13]. These instruments are available in both Sinhalese and Tamil, the main languages spoken in the country. Tamil versions had been validated and previously used in the Tamil-speaking population in the Northern Province [7–13]. New instruments were adapted for use in the study setting through established procedures and field tested during a pilot study conducted at a separate primary care facility in Jaffna district [14].

The sociodemographic section of the questionnaire included 31 variables used in a previous epidemiological cross-sectional study among Tamil populations in Sri Lanka [8]. The conflict and displacement questionnaire was also previously used in Sri Lanka to explore displacement, conflict experience, services available throughout displacement, and experience of return [8]. Depression and anxiety were assessed using the Hopkins Symptom Checklist-25 (HSCL-25). The HSCL-25 has been translated into Tamil and used in a previous study in Northern Sri Lanka [7, 12A]. In concordance with previous research, a cutoff score of 1.75 for each anxiety and depression were used to identify positive cases [7]. Expression of somatoform disorders was assessed using the Patient Health Questionnaire (PHQ-15), where the Tamil language version of the PHQ has been used previously among IDPs and has adequate internal consistency [8, 9].

PTSD was measured using the first section of the Harvard Trauma Questionnaire (HTQ), which has been validated and used in a previous study in Jaffna district [7, 13]. Those that reported a three or a four to at least one question in the recurring symptoms sub-section, at least two questions on the arousal sub-section, and at least three questions in the avoidance sub-section were classified as screening positive for PTSD symptoms [7].

The Psychosis Screening Questionnaire (PSQ) was used to screen for symptoms of psychosis in respondents. The PSQ was previously used in a national prevalence study in Sri Lanka^7^ but not among post-conflict populations, although the PSQ has been used in conflict settings in other countries. Cutoff scores for psychosis were in agreement with previous research and sensitivity analyses were conducted excluding hypomania from this case definition [14, 15].

Lubben Social Network Scale (LSNS-6) and Multidimensional Support Scale (MDSS) were used to measure and assess social networks and support. Both instruments have been previously validated and used in Tamil language among IDP populations in the Northern Province [9, 16]. Using the LNS, participants with scores of 12 or greater were considered to have adequate social networks [17]. Social support availability and adequacy were assessed via the MDSS. As a cut-off point is not standard for this measure, total scores were sorted by tertiles to create availability and adequacy scores [17]. For the section on social support availability, summed scores greater than 32 out of 48 indicated high availability, scores of 17 to 32 indicated moderate availability, and scores of 16 or lower indicated low availability. For social support adequacy, scores of greater than 22 out of 33 indicated high adequacy, scores of 12 to 22 indicated moderate adequacy, and scores of 11 or less indicated low adequacy.

A modified, Sri Lankan version of the Client Service Receipt Inventory (CSRI) was used to record health service utilization aspects [18]. This instrument had been previously translated, back translated and adapted for local context [18]. The modified version used in the current study was piloted before use in the full study to ensure cultural appropriateness and validity.

Data were collected across 25 randomly selected primary care facilities between 20 June and 10 October 2016. All interviews were conducted on-site with data collection forms created using Kobo toolbox, version 1.4.8 [19] and data were hosted at a secure, encrypted server.

Data were downloaded from the secure server to SPSS version 20.0 and cleaned [20]. Data analysis was conducted using SAS version 9.3 [21]. To create a representative sample of all care-seeking individuals in the Northern Province, data were weighted according to total district population size to account for unequal probabilities of selection by district, and respondents from larger districts received larger weights than those from smaller districts. Demographics and prevalence of the five most prevalent MHDs – anxiety, depression, PTSD, expression of somatoform symptoms, and psychosis with hypomania – were obtained using survey means and frequency accounting for two-stage sampling design and sampling weights. Those presenting with any MHD were categorized as screening positive; comorbidities were defined as any combination of screening positive for two MHDs and the number of two-way comorbidities was calculated. Associations between MHDs and social, demographic, economic, and conflict and displacement-related factors were investigated via univariable logistic regression. Multivariable logistic regression considered each MHD individually and included demographics, conflict- and displacement-related factors, social support structures, clinic type and utilization, and participant’s concept of adequacy of care. The final model was selected using backwards selection with a removal threshold of 0.1. Multicollinearity between predictors was assessed using variance inflation factors (VIFs) and correlations.

Informed written consent was obtained and participants were free to withdraw at any time from the study. If participants were identified to have suicidal ideations or serious mental illness, research team members immediately referred them to specialized services following standard operating procedures. Maximum effort was taken to protect privacy during interviews and ensure confidentiality of data collected.

## Results

Out of the 1025 individuals approached, a total of 1017 interviews with individuals were completed, but two persons reported no displacement at any point and were removed from analysis, yielding a final sample of 1015 adults (Fig. 1). More than half of participants were older than 50 years (60.7%; 95% CI, 56.2–65.1%); the mean age was 53.2 years (95% CI, 51.6–54.8%) (Table 1). Approximately half the respondents were female (52.2%; 95% CI, 44.1–60.2%), 79.6% were married (95% CI, 75.8–83.4%), 72.7% were Hindu (95% CI, 60.9–84.5%), and 93.1% were of Tamil ethnicity (95% CI, 88.4–97.9%).

**Table 1:** Prevalence of demographic, socioeconomic, health care utilization, conflict, and displacement related factors

Variable	Level	N	Weighted Percent	95% CI
Age Group	18 to 34	144	15.0	10.3–19.8
	35 to 49	278	24.3	21.7–26.8
	50 to 64	335	32.2	25.9–38.4
	65 +	258	28.5	23.1–33.9
Gender	Female	533	52.2	44.1–60.2
	Male	482	47.8	39.8–55.9
Marital Status	Married	803	79.6	75.8–83.4
	Widowed, Separated, Divorced or Missing	164	15.8	13.2–18.4
	Never Married	48	4.5	2.7–6.4
Ethnicity	Muslim or Sinhala	109	6.9	2.1–11.6
	Tamil	906	93.1	88.4–97.9
Religion	Hindu	680	72.7	60.9–84.5
	Islam	111	7.2	2.4–12.1
	Christian or other	224	20.0	8.2–31.8
Employment Status	Employed	434	38.6	30.1–47.0
	Unemployed/Off Sick/Disabled	101	11.2	7.5–14.8
	Student, Retired, Other	70	8.3	6.5–10.2
	Housewife / At Home	410	41.9	33.8–50.1
Education Level	No formal education, other education	90	8.9	6.6–11.1
	Grades 1-Grade 5	307	27.8	22.6–33.0
	Grades 6 through O/Ls (Grade 12)	511	51.9	46.2–57.6
	University or Higher	107	11.4	8.5–14.3
Household Size	1 to 3	353	34.9	31.6–38.4
	4 to 5	442	41.1	34.9–47.2
	6 to 10	220	23.9	19.9–27.9
Residence Location	Village / Rural Area	854	83.3	76.4–90.2
	City / Town/ Urban Area	86	10.0	3.4–16.6
	Plantation, Coastal, Other	74	6.7	1.5–11.9
Born in Region?	No	135	8.8	6.8–10.8
	Yes	880	91.2	89.2–93.1
Lived in Region for Conflict?	No	117	9.9	7.2–12.6
	Yes	898	90.1	87.4–92.8
Number of Times Displaced	Once	192	22.5	13.4–31.5
	More Than Once	823	77.5	68.5–86.5
Where Were You Displaced?	IDP camp	799	70.2	64.4–75.9
	With Family, Friends, In Own Home	142	29.8	24.0–35.5
Variable	Level	N	Weighted Percent	95% CI
Did you return to your area of origin? If no, why not?	Home/Village Destroyed, Nowhere to go	52	5.3	2.6–8.1
	Did not want to return to old home	79	9.5	5.8–13.2
	Government directed to a different location	35	2.7	1.7–3.7
	Did return to area of origin	848	82.4	76.8–88.0
Were you a combatant during conflict?	No	989	97.8	96.7–98.9
	Yes	26	2.2	1.1–3.3
Were you injured during conflict?	No	820	83.9	81.7–86.2
	Yes	194	16.0	13.8–18.3
Loss of Family During Conflict	No	549	62.1	54.9–69.3
	Yes	465	37.9	30.7–45.0
Injury to Family during Conflict	No	358	43.5	33.1–53.9
	Yes	655	56.5	46.1–66.9
Did you lose property during the conflict? If so, were you able to reclaim it?	Was not able to reclaim properties	547	57.0	50.5–63.6
	Was able to reclaim properties	408	35.7	30.5–40.9
	Did not lose property as a result of the conflict	59	7.3	4.5–10.0
Social Support Availability	Low	395	40.7	34.9–46.4
	Medium	370	34.6	28.6–40.5
	High	250	24.7	22.4–27.0
Social Support Adequacy	Low	268	22.4	16.4–28.3
	Medium	178	17.8	15.0–20.7
	High	568	59.8	53.7–65.9
Social Network Adequacy	Inadequate	231	21.7	17.9–25.5
	Adequate	784	78.3	74.5–82.0
Saw general medical doctor within past 3 months	No	85	7.9	4.4–11.4
	Yes	929	92.1	88.6–95.6
Saw mental health specialist within past 3 months	No	836	82.8	79.2–86.3
	Yes	174	17.2	13.6–20.8
I feel as though health providers don’t understand my problems	Disagree	889	89.6	85.1–94.1
	Agree	125	10.4	5.9–14.9
The care I received was not good	Disagree	864	87.9	83.1–92.8
	Agree	150	12.1	7.2–16.9
Hospital Type	District hospital	933	86.8	62.7–100.0
	Primary medical care unit	82	13.2	0.0–37.3
Variable		N	Weighted Mean	**95% CI**
Days Without Food Within Past Month		960	0.9	0.7–1.1
Age		1015	53.2	51.6–54.8

More than three-quarters of participants reported more than one displacement experience (77.5%; 95% CI, 68.5–86.5%); 70.2% reported displacement to an IDP camp (95% CI, 64.4–75.9%). The majority (82.4%) of participants reported returning to areas of origin post-displacement, but 5.3% (95% CI, 2.6–8.1%) reported nowhere to return, primarily due to destruction of home or village. More than half of respondents (57.0%; 95% CI, 50.5–63.6%) reported inability to recover property lost during the conflict while only 7.3% (95% CI, 4.5–10.0%) reported not having lost property. Of respondents, 16.0% reported injuries to oneself during conflict (95% CI, 13.8–18.3%), 37.9% reported loss of family (95% CI, 30.7–45.0%) and 56.5% reported injury to family during conflict (95% CI, 46.1–66.9%).

More than half of respondents (58.8%; 95% CI, 53.8–62.3%) screened positive for any MHD based on determined cutoffs (Table 2). Furthermore, 42.4% (95% CI, 38.6–46.1%) of respondents screened positive for two or more MHDs.

**Table 2:** Prevalence of mental health disorders

Variable	N	Weighted Percent	95% CI
Any Mental Health Disorder	635	58.8	53.8–62.3
Two Mental Health Disorders	455	42.4	38.6–46.1
Psychosis (with hypomania)	187	17.6	13.3–21.9
Positive Screen for Anxiety	497	46.7	41.9–51.5
Positive Screen for Depression	454	41.6	38.7–44.5
Positive Screen for PTSD	863	13.7	10.6–16.8
Positive Screen for Expression of Somatoform Symptoms	300	27.6	23.6–31.5

The prevalence of anxiety and depression were 46.7% (95% CI, 41.9–51.5%) and 41.6% (95% CI, 38.7–44.5%), respectively. Prevalence of PTSD was 13.7% (95% CI, 10.6–16.8%), expression of somatoform symptoms was 27.6% (95% CI, 23.6–31.5%), and psychosis with hypomania was 17.6% (95% CI, 13.3–21.9%).

For those screening positive for anxiety, females had 3.82 (95% CI, 1.1–8.5) times the odds of males; those unemployed had 3.3 (95% CI, 1.9–5.7) times the odds of those employed; those that reported loss of or injury to family had 1.7 (95% CI 1.1–2.7) and 2.0 (95% CI, 1.4–2.9) times the odds of those who did not report loss or injury, respectively; and those that reported low social support, inadequate social networks, low or medium adequacy of social support had 1.7 to 2.4 times the odds of those who reported high social support, adequate networks or high adequacy of support (Table 3). Education was strongly related to anxiety odds; those with lower education levels reported higher odds of anxiety compared with those with university level education. Only having seen a mental health specialist in the past three months was related to lower odds of anxiety (OR, 0.2; 95% CI, 0.1–0.5).

**Table 3:** Demographic, socioeconomic, health care utilization, conflict, and displacement related factors associated with mental health disorders, multivariable logistic regression models

Variable^a,b^	Level	Anxiety (*N* = 463)		Depression (*N* = 422)		PTSD (*N* = 143)		Somatoform (*N* = 278)^d^		Psychosis (*N* = 172)^e^	
		aOR^c^ (95% CI)	*p*-value	aOR^c^ (95% CI)	*p*-value	aOR^c^ (95% CI)	*p*-value	aOR^c^ (95% CI)	*p*-value	aOR^c^ (95% CI)	*p*-value
Age Group	18 to 34	0.4 (0.1–1.6)	0.0035							**0.4 (0.2–0.9)**	0.0035
	35 to 49	1								1	
	50 to 64	0.9 (0.5–1.7)								0.7 (0.4–1.1)	
	65 +	0.6 (0.3–1.3)								**0.5 (0.3–0.8)**	
Gender	Female	**3.8 (1.7–8.5)**	0.0011					**2.2 (1.2–3.9)**	0.0072		
	Male	1						1			
Ethnicity	Muslim or Sinhala			**0.1 (0.0–0.4)**	0.0022	**0.0 (0.0–0.6)**	0.0231				
	Tamil			1		1					
Religion	Hindu			1	0.0372	1	0.0272				
	Islam			**6.7 (1.4–32.2)**		8.3 (0.6–122.0)					
	Christian or other			1.1 (0.7–1.7)		0.6 (0.4–1.1)					
Employment Status	Employed	1	< 0.0001	1	< 0.0001	1	0.068	1	0.0017		
	Unemployed/Off Sick/Disabled	**3.3 (1.9–5.7)**		**4.2 (2.5–7.2)**		0.8 (0.3–1.9)		**2.8 (1.0–7.4)**			
	Student, Retired, Other	0.7 (0.3–1.5)		0.6 (0.2–2.1)		**1.9 (1.1–3.3)**		1.0 (0.3–3.0)			
	Housewife / At Home	1.0 (0.5–2.0)		**2.1 (1.4–3.2)**		1.6 (0.6–4.0)		1.2 (0.6–2.2)			
Education Level	No formal education, other education	**2.4 (1.1–5.4)**	0.0067	0.6 (0.2–2.1)	< 0.0001	**1.9 (1.1–3.3)**	0.0395	0.8 (0.3–1.8)	0.0123		
	Grades 1-Grade 5	**3.0 (1.5–5.8)**		**2.2 (1.3–3.7)**		**4.0 (1.4–11.3)**		1.5 (0.9–2.8)			
	Grades 6 through O/Ls (Grade 12)	**1.9 (1.1–3.5)**		2.6 (0.8–8.7)		2.3 (0.8–6.5)		**1.9 (1.2–3.0)**			
	University or Higher	1		1		1		1			
Household Size	1 to 3	1	< 0.0001								
	4 to 5	0.9 (0.5–1.5)									
	6 to 10	**2.1 (1.2–3.8)**									
Born in Region?	No			1	0.0049	1	0.0136	1	0.0185		
	Yes			**0.4 (0.2–0.8)**		**0.4 (0.2–0.8)**		**0.5 (0.3–0.9)**			
Lived in Region for Conflict?	No									1	0.0451
	Yes									**3.8 (1.0–14.0)**	
Variable^a,b^	Level	Anxiety (N = 463)		Depression (N = 422)		PTSD (N = 143)		Somatoform (N = 278)^d^		Psychosis (N = 172)^e^	
		aOR^c^ (95% CI)	p-value	aOR^c^ (95% CI)	p-value	aOR^c^ (95% CI)	p-value	aOR^c^ (95% CI)	p-value	aOR^c^ (95% CI)	*p*-value
Number of Times Displaced	Once					1	0.0952				
	More Than Once					1.7 (0.9–3.1)					
Did you return to your area of origin? If no, why not?	Home/Village Destroyed, Nowhere to go			1.6 (0.8–3.2)	0.0186						
	Did not want to return to old home			1.6 (0.8–3.3)							
	Government directed to a different location			2.7 (0.8–8.6)							
	Did return to area of origin			1							
Loss of family during conflict	No	1	0.0026	1	0.0029	1	0.0737	1	0.0095		
	Yes	**1.7 (1.1–2.7)**		**2.0 (1.3–3.0)**		1.5 (1.0–2.4)		**1.6 (1.1–2.2)**			
Injury to family during conflict	No	1	0.0001	1	0.0485	1	0.0824	1	0.0005	1	< 0.0001
	Yes	**2.0 (1.4–2.9)**		**1.9 (1.0–3.7)**		2.1 (0.9–4.7)		**2.3 (1.5–3.8)**		**1.7 (1.3–2.2)**	
Social Support Availability	Low	**2.4 (1.2–4.8)**	< 0.0001							1.8 (1.0–3.5)	0.0116
	Medium	1.0 (0.5–2.0)								0.9 (0.4–2.4)	
	High	1								1	
Social Support Adequacy	Low	**2.1 (1.3–3.4)**	< 0.0001	**3.7 (2.6–5.2)**	< 0.0001	**8.9 (4.2–18.8)**	< 0.0001	**4.2 (2.3–7.4)**	< 0.0001	**1.9 (1.0–3.7)**	0.0265
	Medium	**2.7 (1.8–4.2)**		**3.0 (1.3–6.9)**		**7.1 (3.3–15.2)**		**3.6 (2.0–6.5)**		**2.4 (1.2–4.8)**	
	High	1		1		1		1		1	
Social Network Adequacy	Inadequate	**1.7 (1.3–2.3)**	0.0007	**1.9 (1.2–2.9)**	0.0066	1.5 (1.0–2.4)	0.0796	**1.9 (1.1–3.4)**	0.0314		
	Adequate	1		1		1		1			
Saw MH specialist within past 3 months^f^	No	1	0.0002	1	< 0.0001			1	< 0.0001	1	0.0145
	Yes	**0.2 (0.1–0.5)**		**0.2 (0.1–0.4)**				**0.2 (0.1–0.4)**		**0.5 (0.2–0.9)**	
The care I received was not good	Disagree			1	0.0141						
	Agree			**1.8 (1.1–2.8)**							
Hospital Type	District Hospital							1	0.0159		
	Primary medical care unit							**0.6 (0.4–0.9)**			
Days without food in the past month				**1.2 (1.1–1.2)**	< 0.0001			**1.1 (1.1–1.2)**	0.0002		

^a^Empty cells in the table are due to lack of significance at the α = 0.1 level in the multivariable model for that disorder. ^b^ Bold face signifies significance at the α = 0.05 level

^c^aOR: adjusted Odds Ratio – the odds ratio obtained from the multivariable model. ^d^ Somatoform stands for somatoform symptoms.^e^ Psychosis refers to psychosis with hypomania. ^f^MH: Mental Health; Values of “1” in the table represent the reference category for that variable

Unemployment was also significantly related to higher odds of depression (OR, 4.2; 95% CI, 2.5–7.2) compared with those employed; similarly, housewives or those who stayed at home reported higher odds of depression (OR, 2.1; 95% CI, 1.4–3.2). Loss of, or injury to, family during conflict were both associated with nearly double the odds of depression compared with those who did not lose or have family injured (OR, 2.0; 95% CI, 1.3–3.0 and OR, 1.9; 95% CI, 1.0–3.7, respectively). Further, those reporting low or medium social support adequacy or inadequate social networks had 1.9–3.7 times the odds of depression versus those who reported high social support adequacy and adequate social networks. For each one-day increase without food in the past month, odds of depression increased by 15.6% (95% CI, 1.1–1.2). Those who saw a mental health expert in the past three months had significantly lower odds of depression compared with those who did not (OR, 0.2; 95% CI, 0.1–0.4).

Those with less education had higher odds of PTSD and those who reported low or medium levels of social support adequacy had 8.9 (95% CI, 4.2–18.8) or 7.1 (95% CI, 3.3–15.2) times the odds of PTSD, respectively compared with those who reported high social support adequacy. Those who were of Sinhala ethnicity compared with Tamil ethnicity, and those born in the region compared with born elsewhere had significantly lower odds of PTSD.

For persons presenting with somatoform symptoms exceeding the threshold, females had 2.2 (95% CI, 1.2–3.9) times the odds compared with males, and those unemployed, sick, or disabled had 2.8 (95% CI, 1.0–7.4) times the odds of those that were employed. Those that reported loss of, or injury to, family had 1.6 (95% CI, 1.1–2.2) and 2.3 (95% CI, 1.5–3.8) times the odds of those who did not report loss or injury, respectively**.** Having low or medium social support adequacy was associated with 4.2 (95% CI, 2.3–7.4) and 3.6 (95% CI, 2.0–6.5) times the odds of presenting with somatoform symptoms compared with those reporting high social support adequacy, while those reporting inadequate social networks had 1.9 (95% CI, 1.1–3.4) times the odds of those reporting adequate networks. Each one-day increase without food in the past month was associated with a significant 11.1% increase in odds of presenting with somatoform symptoms (95% CI, 1.1–1.2). Those who were born in the region and those who saw a mental health expert in the past three months had 0.5 (95% CI, 0.3–0.9) and 0.2 (95% CI, 0.1–0.4) lower odds of presenting with somatoform symptoms, respectively.

Those who lived in the region during conflict had 3.8 (95% CI, 1.0–14.0) times the odds of having psychosis with hypomania when compared with those who did not. Similarly, those who had family injured during the conflict had 1.7 (95% CI, 1.3–2.2) times the odds of having psychosis when compared with those who did not. Low and medium social support adequacy levels was associated with 1.9 (95% CI, 1.0–3.7) and 2.4 (95% CI, 1.2–4.8) times the odds of psychosis, respectively, when compared with those who reported high levels of social support adequacy. Those who had seen a mental health expert in the past three months had significantly lower odds of psychosis when compared with those who had not (OR, 0.5; 95% CI, 0.2–0.9).

## Discussion

Our study provides the first comprehensive overview of mental health disorder prevalence among adult, primary care attendees in the post-conflict region of the Northern Province, Sri Lanka. The primary aim of Phase One of COMGAP-S was met; this study collected data from 1015 adults attending 25 primary care settings in post-conflict Northern Province, Sri Lanka and investigated prevalence of MHDs. The study found high proportions of depression, anxiety, PTSD, expression of somatoform symptoms and psychosis with hypomania among primary care attendees (41.6, 46.7, 13.7, 27.6, and 17.6% respectively). Similar studies conducted in post-conflict Sri Lanka showed lower prevalence rates than found in our study. One study conducted with post-conflict primary care attendees at 16 facilities in Northern Province reported prevalence of major depression of 4.5% [5]. At a population level, one study reported 22.2% of depression, 32.6% of anxiety, and 7.0% of PTSD in a post-conflict community in Northern Province [7]. Two studies among post-conflict IDPs in Northern Province reported symptoms of psychosis at 9.7% [10], major depression at 5.1%, and anxiety at 1.3%, PTSD at 2.8% and somatoform disorders at 14.0% [8]. Past prevalence rates reported in the introduction section originate from studies conducted after the end of conflict on a narrow scope of mental health conditions in a limited number of settings. The variance in these rates could have occurred due to the following factors: different assessment tools utilized, various study design types, limited settings geographic location of clinics to conflict area. The higher prevalence rates found in our study could reflect the experience of many in Northern Province who lived through prolonged displacement along with exposure to natural disasters and conflict [5, 7, 8, 10].

The present study found factors such as female gender, unemployment, low education level, loss of, or injury to family during conflict, low social support, and inadequate social networks were significantly associated with increased odds of screening positive for MHDs. Being female increased odds of screening positive for anxiety and somatoform symptoms, supporting previous research conducted in Sri Lanka both at community and primary care level [5, 7]. Participants who indicated they were unemployed had increased odds of screening positive for anxiety, depression, and expression of somatoform symptoms, and being a student or retired increased odds of screening positive for PTSD, supporting work conducted with IDPs in Sri Lanka [6]. Lower levels of formal education also appeared to significantly increase odds of screening positive for symptoms of anxiety, depression, and PTSD supporting previous work [6]. Having lived in the region during conflict appeared significantly associated with increased odds of screening positive for psychosis with hypomania and is supported by previous work in the country [10].

Experiences associated with conflict, specifically injury to, or loss of, family significantly increased odds of screening positive for anxiety, depression, expression of somatoform symptoms, and psychosis with hypomania. Findings indicate that low availability of social support and injury to family during conflict consistently increased odds of screening positive for all examined MHDs, with the exception of PTSD. This may be associated with disruption of traditional family structures during conflict, and difficulties experienced in return migration in recent years [6].

Food insecurity was a predictor for screening positive for depression and expression of somatoform symptoms. This could be linked to unemployment rates as households may struggle to find sufficient food adding burden and stress. This is supported by work with IDPs in Sri Lanka, which found food insecurity was as a predictor of mental illness [6].

Our findings support earlier work conducted in Sri Lanka, however this is the first study to screen widely for MHDs, social support/networks, and associations between MHDs and socioeconomic factors at the primary care level. Knowledge gained indicates unmet need at the primary care level and valuable information on associations between MHDs and social and socioeconomic factors. These results signpost the importance of addressing unmet mental health needs of primary care attendees and the necessity of Phase Two implementation: to train primary care practitioners using a scaled-up intervention based on WHO mhGAP. Together with findings from Phase Two implementation, which is currently underway, these results have the potential to support further developments of the mental health policy of Sri Lanka, particularly with regards to the Northern Province. Due to the focus on conflict-affected populations at the primary care level, the study sheds light on the needs of service planning in post-conflict areas.

While the majority of the screening tools chosen were previously validated in Sri Lanka, some were culturally adapted and piloted in this study. Further, while this study used standardized definitions of examined mental disorders, the authors acknowledge there can be debate around trans-cultural conceptions of mental health and distress. While this could have impacted responses, and consideration of mental health constructs deserves debate, it should be noted that Sri Lanka has used European models of health since the nineteenth Century [9].

This study recruited those attending primary care facilities and as this is often the first point of contact for those seeking mental health services in the region [5, 6], prevalence rates could have been overestimated; however, results still show a significant unmet need in the Province. Since this study focused on displaced persons only, findings may not be representative of the overall population; however, weights were used to ensure that the study was representative of all displaced persons in the region. Additionally due to logistical and financial constraints, this study utilized screening tools, which have high sensitivity to detect potential indicators of MHDs, while diagnostic tools are designed with high specificity to establish actual presence of a MHD. Finally, as our protocol required exclusion of persons who had mental disorders too severe to complete the questionnaire the true mental health prevalence could have been slightly higher than reported. However, in the study, no persons were excluded due to this criterion so we do not anticipate differences in prevalence.

## Conclusions

In conclusion, this study reports the prevalence of MHDs, including depression, anxiety, PTSD, psychosis with hypomania, and expression of somatoform symptoms in primary care attendees returned since the end of conflict to the Northern Province, Sri Lanka. Our findings indicate there is high prevalence of mental health disorder and high unmet need for access to mental health services within primary care in the region. The next phase of this study aims to utilize these findings to strengthen the primary care workforce to integrate mental health services into the primary care level and reduce the treatment gap for those in need. Strengthening support at the primary care level is vital for prevention, early detection, and to ensure treatment is delivered in an accessible and appropriate manner. Our study findings will guide Phase 2 of this study: to integrate mental health services into primary care by training the primary care workforce using a scaled-up and culturally adapted version of mhGAP 2.0.

## Abbreviations

COMGAP-S: Integrating mental health into primary care for conflict-affected populations in Northern Sri Lanka
DEFF: Design effect
HSCL-25: Hopkins Symptom Checklist-25
HTQ: Harvard Trauma Questionnaire
IDP: Internally displaced person
LSN: Lubben Social Network Scale
MDSS: Multidimensional Support Scale
MHD: Mental health disorder
mhGAP-ID 2.0: Mental Health Gap Action Plan – Intervention Guide version 2.0
PHQ-15: Patient Health Questionnaire
PSQ: Psychosis Screening Questionnaire
PTSD: Post-Traumatic Stress Disorder
VIF: Variance inflation factor

## References

[1] Porter M, Haslam N (2005). Predisplacement and postdisplacement factors associated with mental health of refugees and internally displaced persons: a meta-analysis. JAMA.

[2] Steel Z, Chey T, Silove D, Marnane C, Bryant R, van Ommeren M (2009). Association of torture and other potentially traumatic events with mental health outcomes among populations exposed to mass conflict and displacement: a systematic review and meta-analysis. JAMA.

[3] Roberts B, Browne, J. A systematic review of factors influencing the psychological health of conflict-affected populations in low- and middle-income countries. Global Public Health: An International Journal for Research Policy and Practice 2011; 6(8): 814—829. 10.1080/17441692.2010.511625 20859816

[4] Siriwardhana C, Wickramage K (2014). Conflict, forced displacement and health in Sri Lanka: a review of the research landscape. Conflict & Health.

[5] Senarath U, Wickramage K, Peiris SL (2014). Prevalence of depression and its associated factors among patients attending primary care settings in the post-conflict Northern Province in Sri Lanka: a cross-sectional study. BMC Psychiatry.

[6] Siriwardhana C, Adikari A, Van Bortel T, McCrone P, Sumathipala A (2013). An intervention to improve mental health care for conflict-affected forced migrants in low-resource primary care settings: a WHO MhGAP-based pilot study in Sri Lanka (COM-GAP study). Trials.

[7] Husain F, Anderson M, Cardozo BL, et al. Prevalence of war-related mental health conditions and association with displacement status in postwar Jaffna District, Sri Lanka JAMA 2011; 306(5): 522—531. 10.1001/jama.2011.1052 21813430

[8] Siriwardhana C, Adikari A, Pannala G, Siribaddana S, Abas M, Sumathipala A, Stewart R (2013). Prolonged internal displacement and common mental disorders in Sri Lanka: the COMRAID study. PLoS One.

[9] Siriwardhana C, Adikari A, Pannala G (2015). Changes in mental health prevalence among long-term displaced and returnee forced migrants in Sri Lanka (COMRAID-R). BMC Psychiatry.

[10] Keraite A, Sumathipala A, Siriwardhana C, Morgan C, Reininghaus U. Exposure to conflict and disaster: a national survey on the prevalence of psychotic experiences in Sri Lanka. Schizophr Res 2016; 171(1): 79—85. 10.1016/j.schres.2016.01.026 26817400

[11] KISH, L. Survey sampling. London, John Wiley & Sons, 1965.

[12] Northern Provincial Council. Statistical Information of the Northern Provincial Council 2014.

[13] Suraweera C, Hanwella R, Sivayokan S, de Silva V. Rating scales validated for Sri Lankan populations. Sri Lanka Journal of Psychiatry 2013; 4(2): 16–24.

[14] Weeks, A., Swerissen, H., & Belfrage, J. (2007). Issues, challenges, and solutions in translating study instruments. Eval Rev, 31(2), 153–165. 10.1177/0193841X06294184 17356181

[15] Soosay I, Silove D, Bateman-Steel C, Steel Z, Bebbington P, Jones PB, Chey T (2012). Trauma exposure, PTSD and psychotic-like symptoms in postconflict Timor Leste: an epidemiological survey. BMC Psychiatry.

[16] Siriwardhana C, Abas M, Siribaddana S, Sumathipala A, Stewart R. Dynamics of resilience in forced migration: longitudinal associations with mental health in a conflict-affected population. BMJ Open 2015a; 5: e006000. 10.1136/bmjopen-2014-006000 PMC4336461 25687898

[17] Lubben J, Blozik E, Gillmann G, Iliffe S, von Renteln Kruse W, Beck JC, Stuck AE (2006). Performance of an abbreviated version of the Lubben social network scale among three European community-dwelling older adult populations. The Gerontologist.

[18] Chisholm D, Sekar K, Kishore Kumar K, Saeed K, James S, Mubbashar M, Srinivasa Murthy R. Integration of mental health care into primary care: demonstration cost-outcome study in India and Pakistan. Br J Psychiatry 2000; 176(6): 581–588. 10.1192/bjp.176.6.581 10974966

[19] Harvard Humanitarian Initiative KoboCollect 1.4.8 (1057), part of Kobo Toolbox 2015.

[20] SPSS Inc. Released 2016. SPSS for windows, version 20.0. Chicago, SPSS Inc.

[21] SAS Institute, Cary, NC.

